# Supplementary figures and images for: Manganese acquisition is essential for virulence of Enterococcus faecalis
Source: PLoS Pathog. 2018 Sep 20;14(9):e1007102. doi: 10.1371/journal.ppat.1007102 (PMC6147510; doi:10.1371/journal.ppat.1007102)

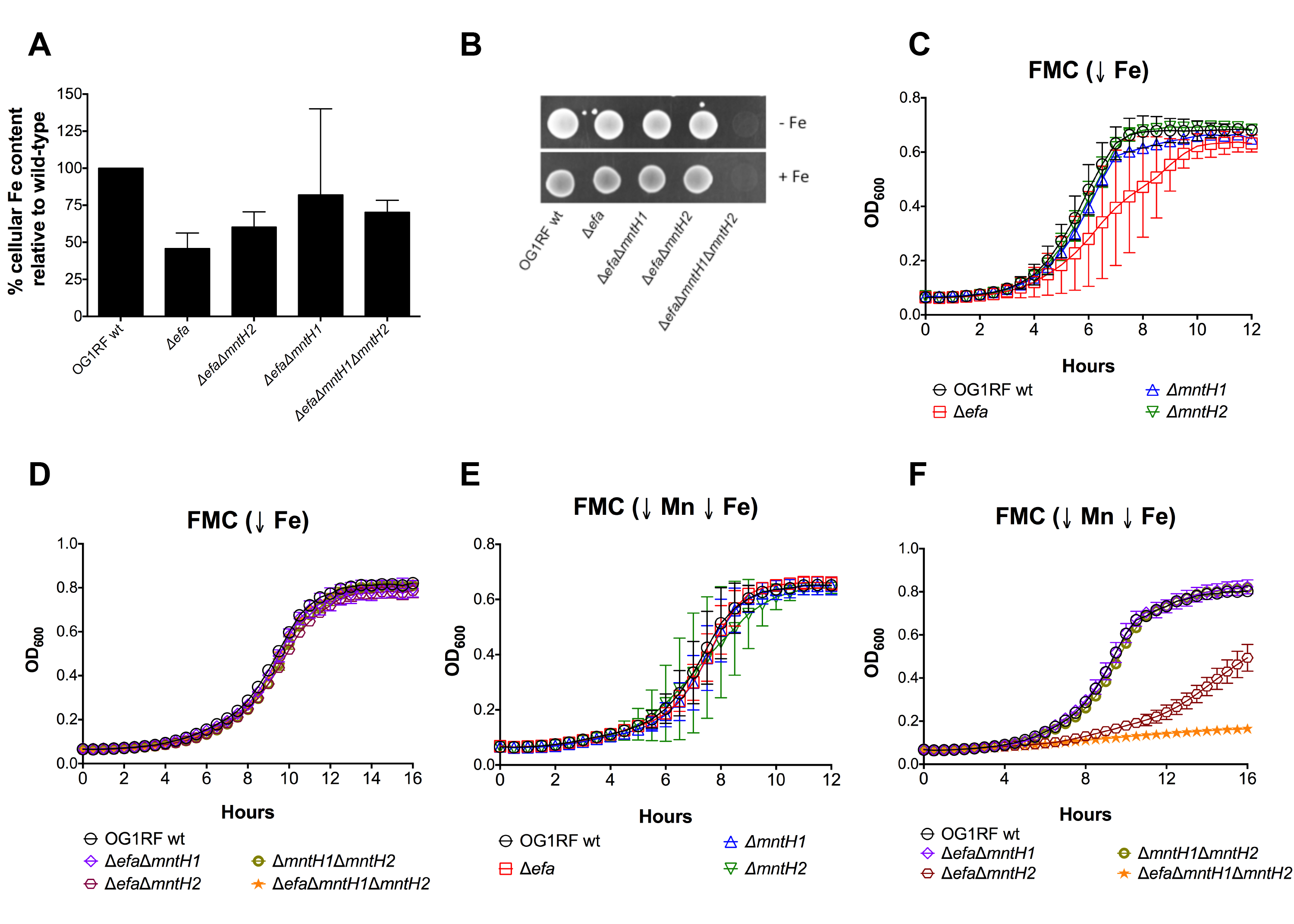

Supplement: S1 Fig — (A) Cellular Fe quantifications of E. faecalis OG1RF with single Δefa, double ΔefaΔmntH1, ΔefaΔmntH2, and triple ΔefaΔmntH1ΔmntH2 mutant strains. Strains were grown to mid-log phase (OD600 ~ 0.5) in BHI prior to analysis. The bar graphs show the relative average Fe content of mutant strains relative to the wild-type and the standard deviations of three independent ICP-OES analyses. (B) Growth of OG1RF (wild-type), Δefa, ΔmntH1, ΔmntH2, ΔefaΔmntH1, ΔefaΔmntH2, ΔmntH1ΔmntH2 and ΔefaΔmntH1ΔmntH2 strains on BHI plates with or without 150 μM FeSO4 supplementation. Overnight cultures were washed, diluted in PBS and 5 μl aliquots spotted on plates. Plates were incubated for 24 hours before being photographed. (C–F) Growth of OG1RF and its derivatives in Fe-depleted (↓ Fe, panels A–B), and Fe- and Mn-depleted (↓ Mn ↓ Fe, panels C–D) FMC. Cells were grown to OD600 ~ 0.2 in complete FMC and diluted 1:100 in either complete FMC or FMC depleted for Fe, or Fe and Mn. Growth was monitored using a Bioscreen growth reader monitor. The graphs show the average and standard deviations of three independent experiments. (TIF) [file ppat.1007102.s001.tif]

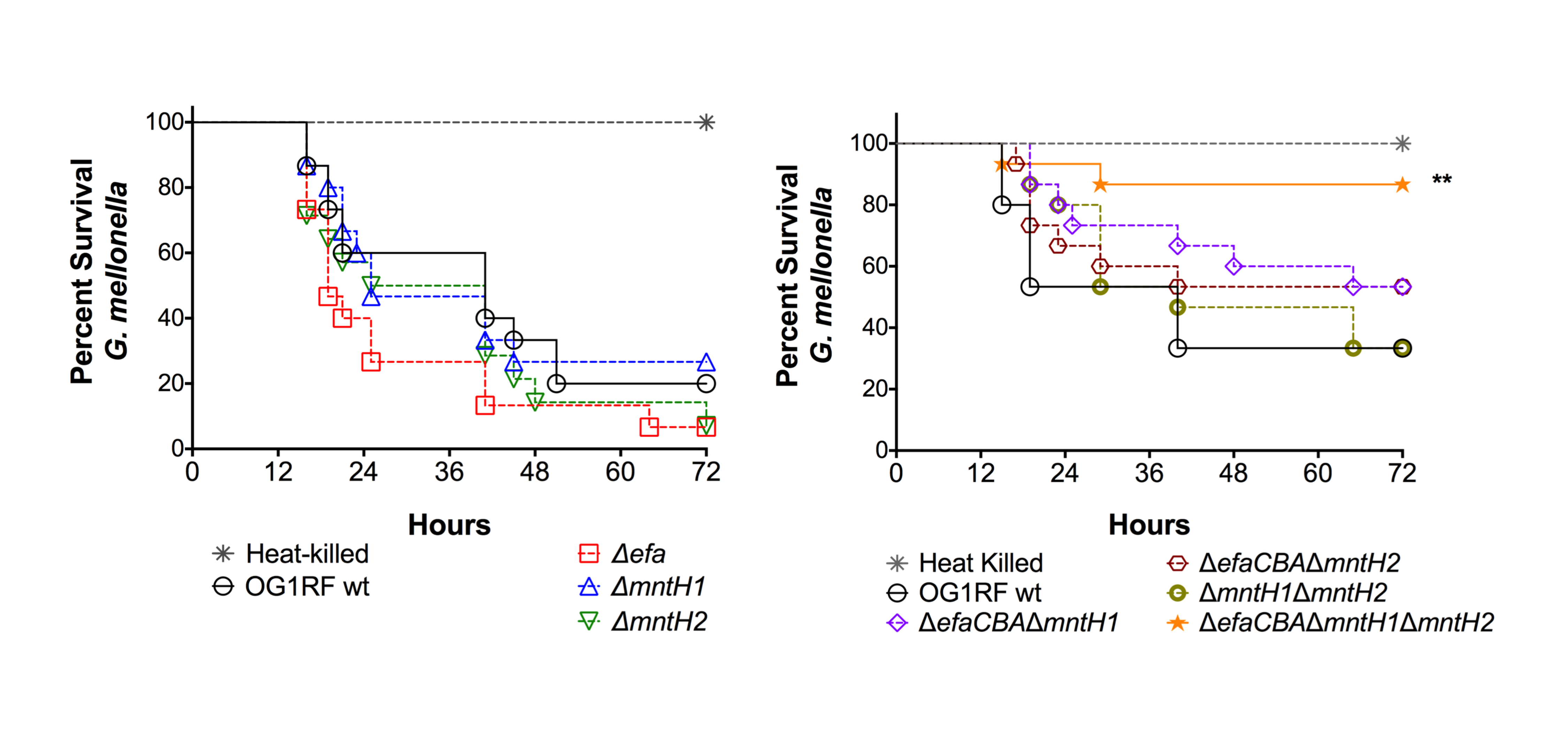

Supplement: S2 Fig — Kaplan-Meyer plots of G. mellonella larvae injected with E. faecalis OG1RF or the single, double, or triple mutants. Each Kaplan-Meyer plot is a representative of an experiment repeated at least six independent times. Differences in the G. mellonella killing rates of the mutant strains compared to the parent OG1RF strain were assessed with the Mantel-Cox log-rank test. (** p ≤ 0.01). (TIF) [file ppat.1007102.s002.tif]

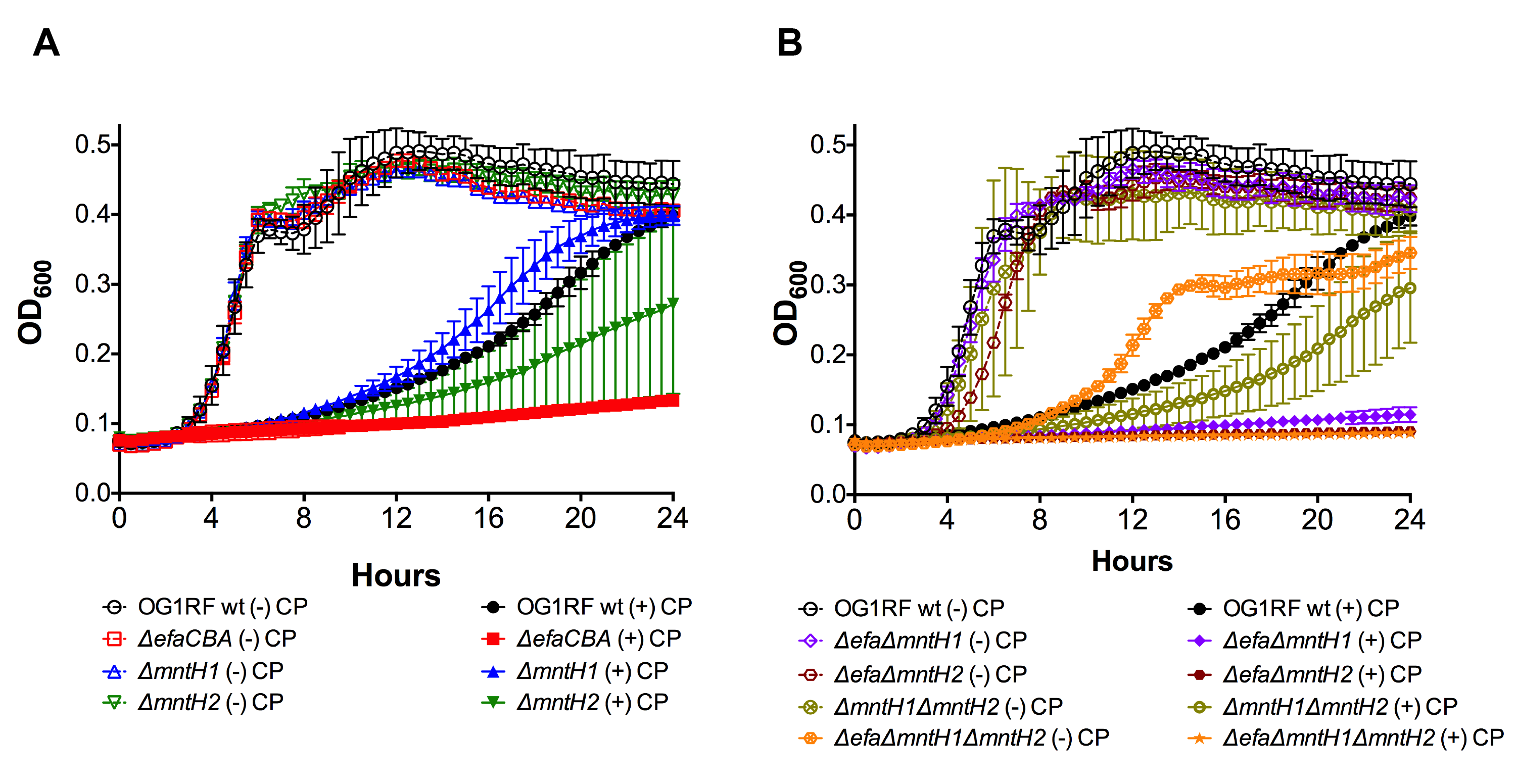

Supplement: S3 Fig — (A–B) Growth curves of single (A), double and triple (B) mutant strains in CP medium in the presence (+ CP) or absence (- CP) of 120 μg ml-1 of native calprotectin. Overnight cultures were diluted 1:50 in BHI and incubated at 37°C for 1 hour prior to diluting 1:100 into CP medium and incubating the strains with calprotectin. Growth was monitored using a Bioscreen growth reader monitor for up to 24 hours. The graphs show the average and standard deviations of three independent cultures. (TIF) [file ppat.1007102.s003.tif]

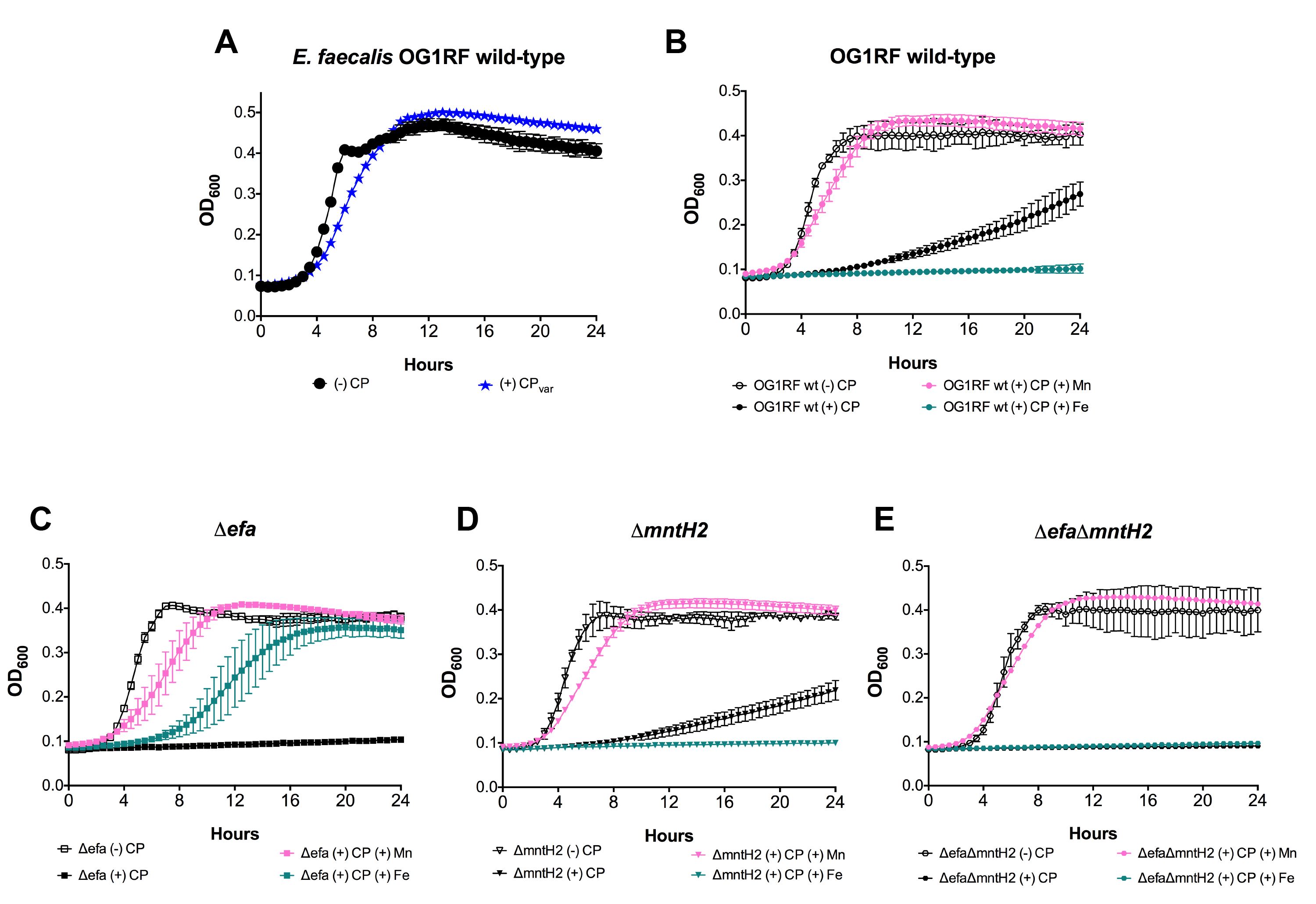

Supplement: S4 Fig — (A) Growth of wild-type OG1RF in the presence of the ΔMn Tail calprotectin variant (+ CPvar) unable to chelate Mn when compared to growth without calprotectin (- CP). (B–E) Growth curves of OG1RF wild-type (B), Δefa (C), ΔmntH2 (D), and ΔefaΔmntH2 (E) strains in the presence of native calprotectin with or without Mn or Fe supplementation. Overnight cultures were diluted 1:50 in BHI and incubated at 37°C for 1 hour prior to diluting 1:100 into CP medium and incubating the strains with 120 μg ml-1 of purified calprotectin. When indicated, 10 μM MnSO4 or FeSO4 was added to the medium. Growth was monitored using a Bioscreen growth reader monitor for up to 24 hours. The graphs show the average and standard deviations of three independent cultures. (TIF) [file ppat.1007102.s004.tif]

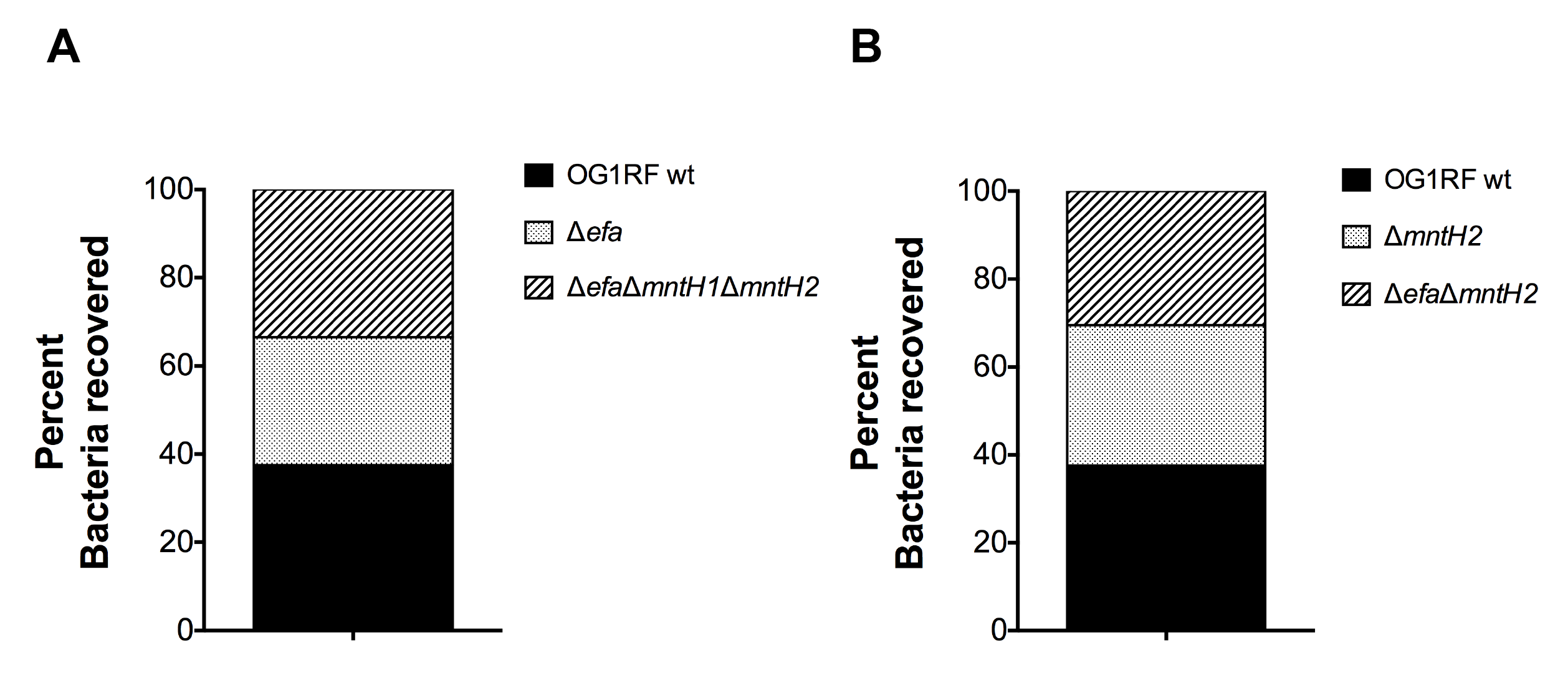

Supplement: S5 Fig — Parent OG1RF was co-cultured for 9 hours with (A) Δefa and ΔefaΔmntH1ΔmntH2, or with (B) ΔmntH2 and ΔefaΔmntH2 strains at an initial inoculum of ~ 2 x 10^3^ CFU (each) in BHI supplemented with 150 μM MnSO4. After 9 hours of incubation, PCR screens were used to determine the frequency with which each individual strain was recovered from the medium. Graphs show the average percent of each strain recovered from two individual experiments. (TIF) [file ppat.1007102.s005.tif]

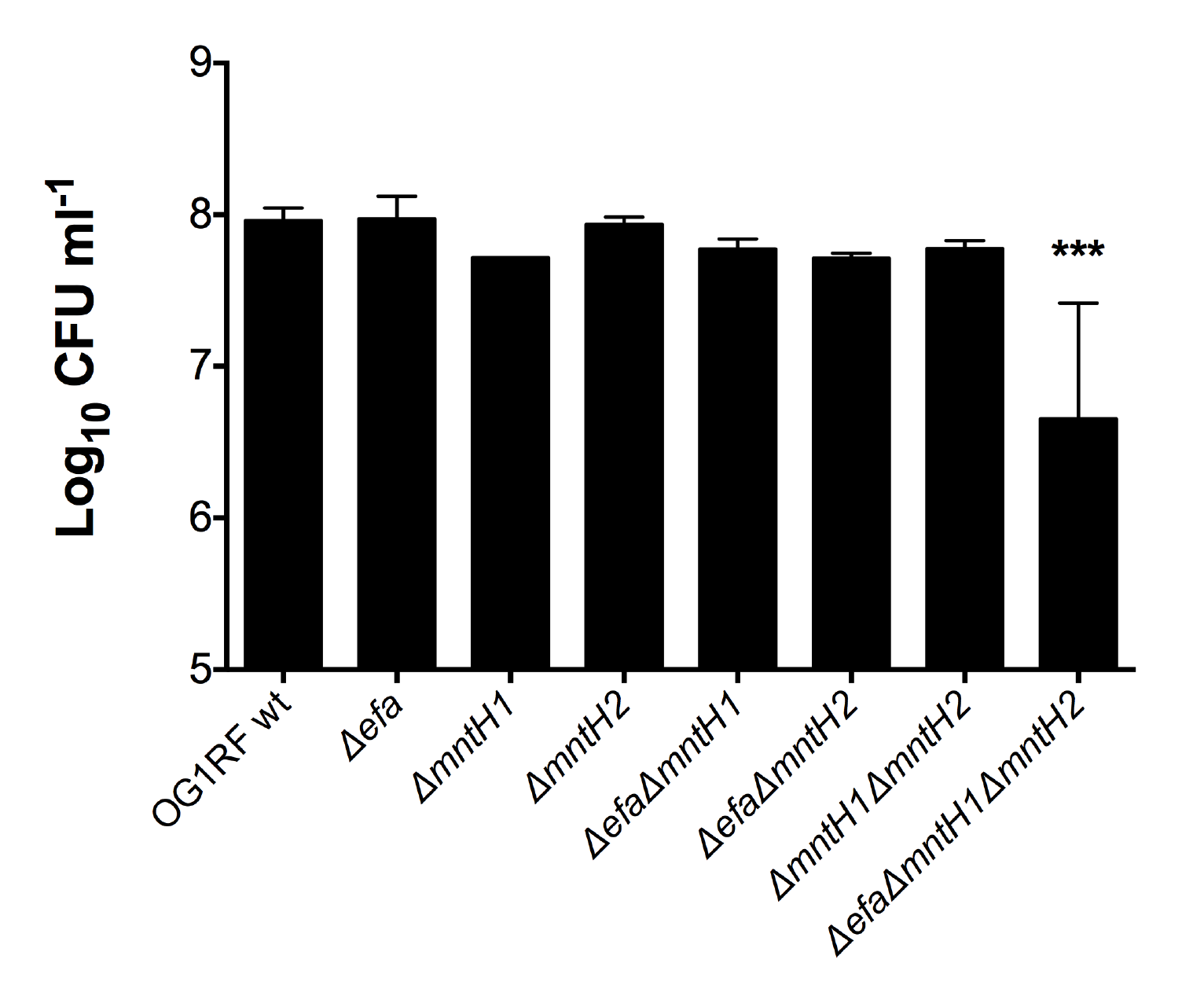

Supplement: S6 Fig — Survival of E. faecalis wild-type (OG1RF) and Mn deletion strains (single Δefa, ΔmntH1, ΔmntH2, ΔefaΔmntH1, ΔefaΔmntH2, ΔmntH1ΔmntH2, ΔefaΔmntH1ΔmntH2) in pooled human urine after 48 hours of incubation. Aliquots at selected time points were serially diluted and plated on BHI + Mn plates for CFU enumeration. The graphs show the average and standard deviations of three independent experiments. (***P ≤ 0.001). (TIF) [file ppat.1007102.s006.tif]

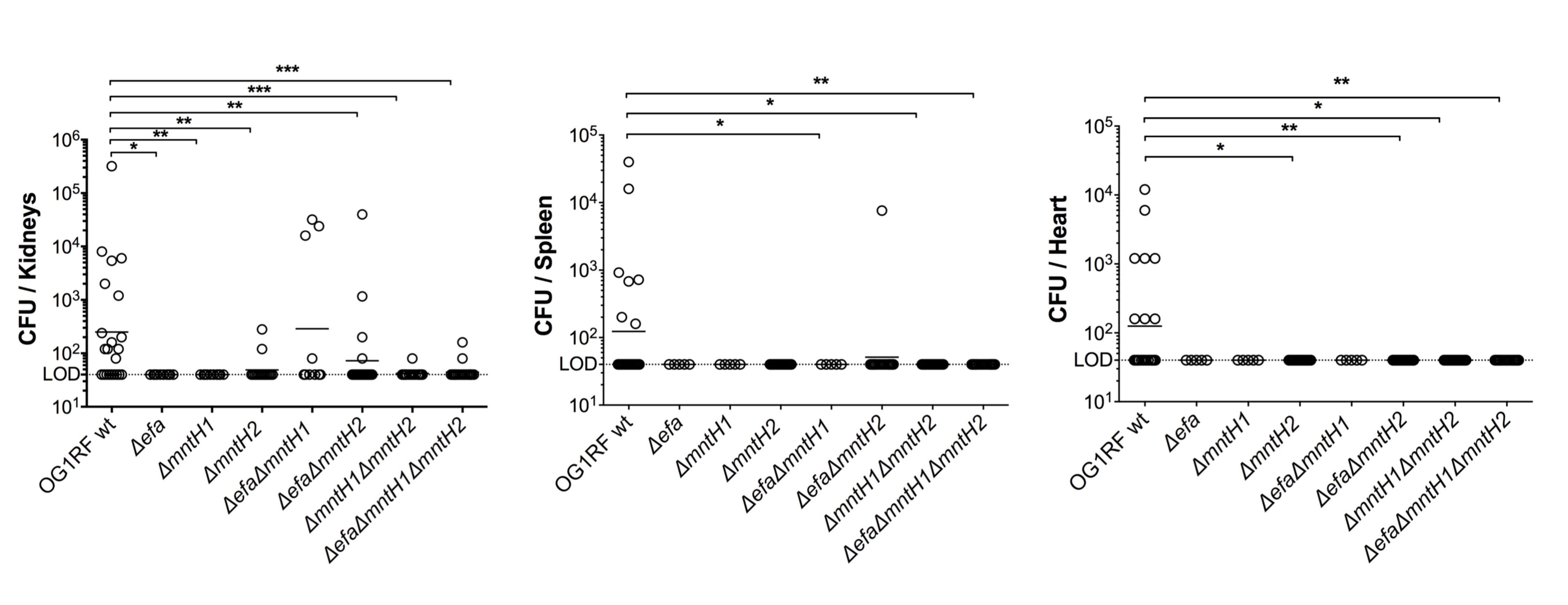

Supplement: S7 Fig — OG1RF and its derivatives were inoculated into the bladder of mice immediately after catheter implantation. After 24 hours, animals were euthanized and bacterial burdens in (A) kidneys, (B) spleens and (C) hearts determined. Graphs show total CFU recovered from these sites, and each symbol represents an individual mouse. Symbols on the dashed line indicate that recovery was below the limit of detection (LOD, 40 CFU). Data were pooled from at least two independent experiments, and median value is shown as a horizontal line. Statistical differences were analyzed by a two-tailed Mann-Whitney U test (*p < 0.05, **p < 0.005, ***p < 0.001). (TIF) [file ppat.1007102.s007.tif]

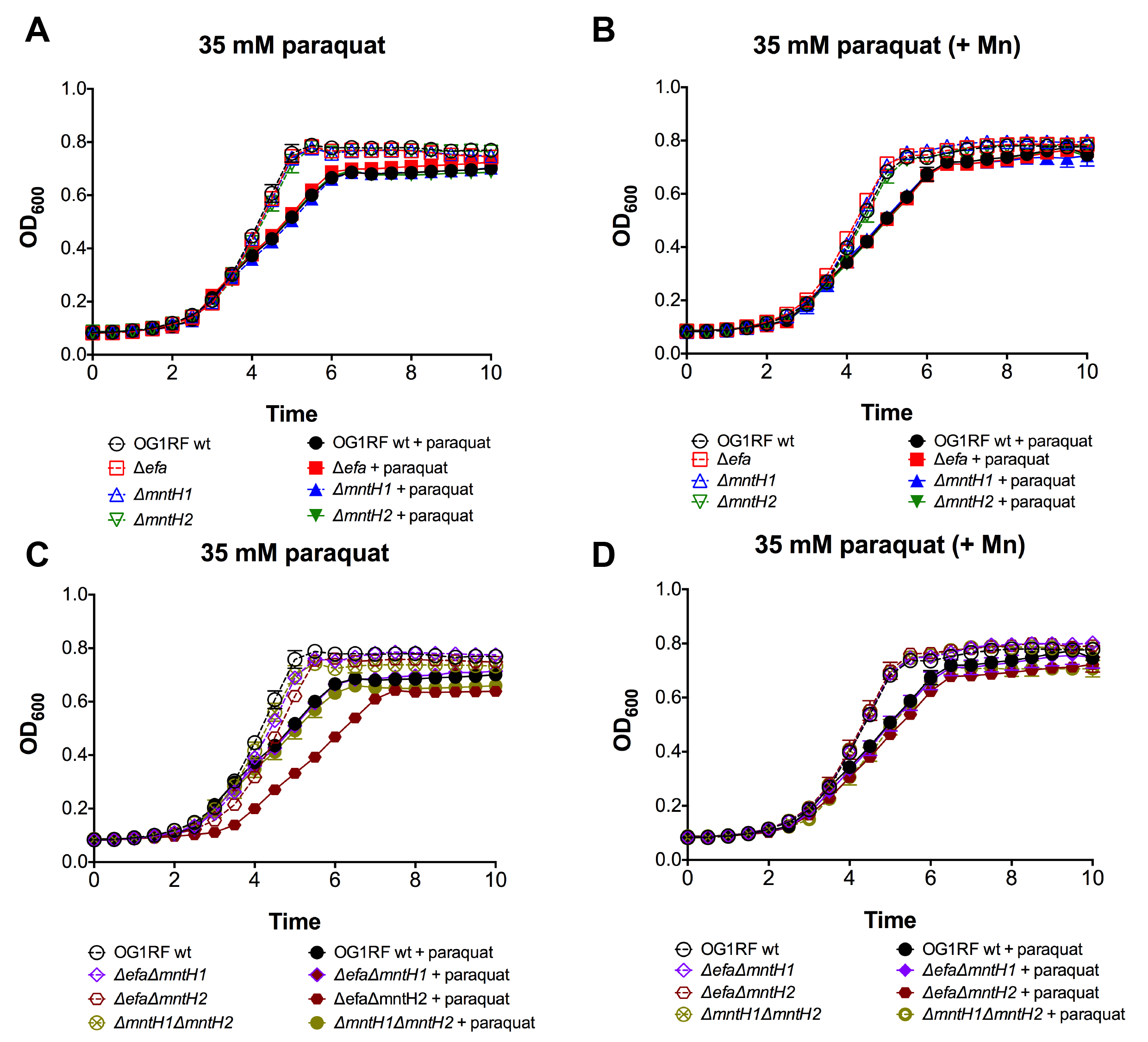

Supplement: S8 Fig — Growth of OG1RF parent, single (A, B) and double (C, D) mutant strains in BHI (A, C) or BHI supplemented with Mn (+ Mn, panels B, D) in the presence of the superoxide generator paraquat. Cells were grown to OD600 ~ 0.2 in BHI and diluted 1:100 in fresh BHI with or without 150 μM MnSO4 and 35 mM paraquat. Growth was monitored using a Bioscreen growth reader monitor. (TIF) [file ppat.1007102.s008.tif]

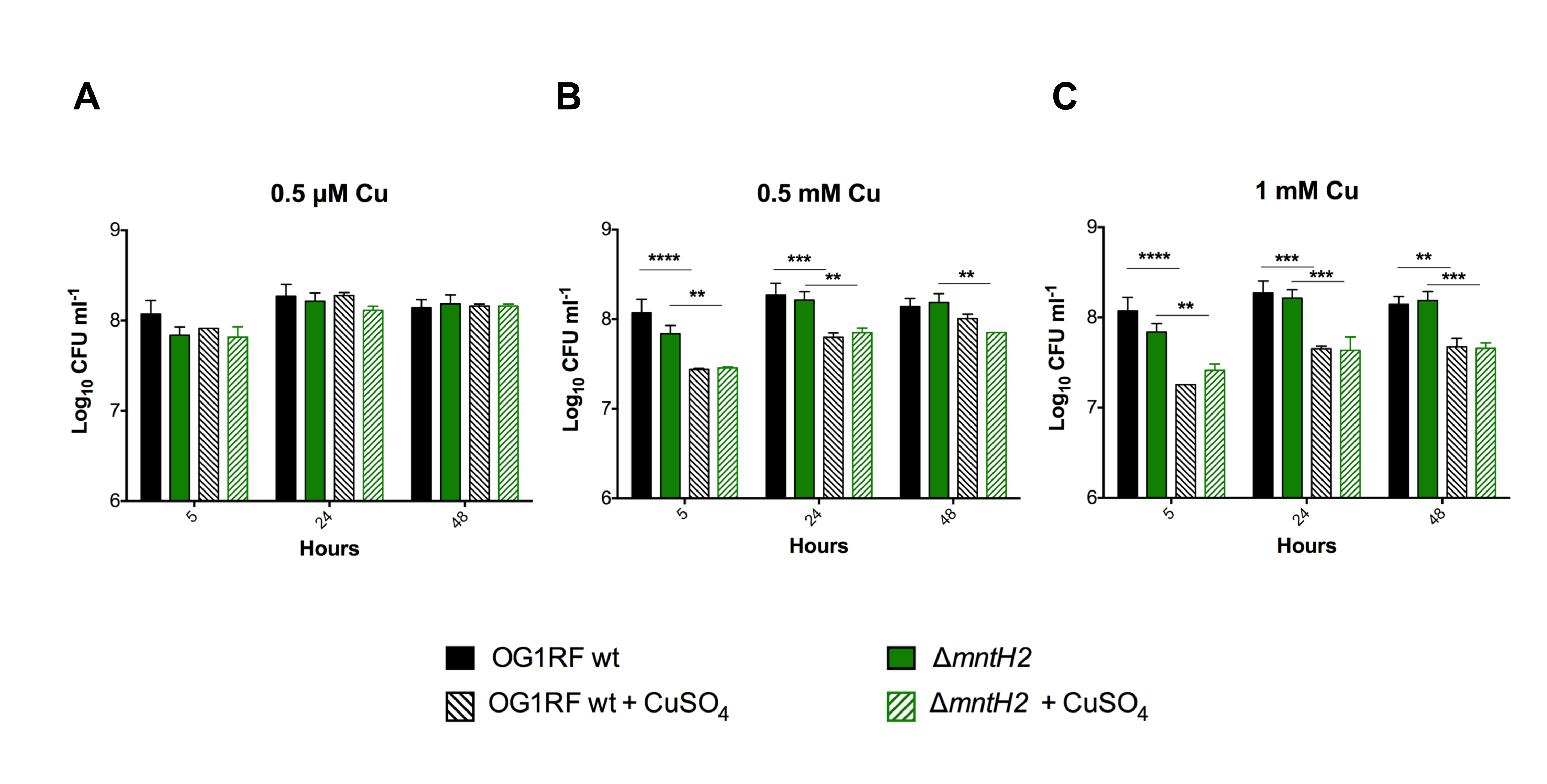

Supplement: S9 Fig — Survival of OG1RF wild-type and ΔmntH2 strains in human urine supplemented with (A) 0.5 μM, (B) 0.5 mM, or (C) 1 mM CuSO4. Control corresponds to urine without CuSO4 supplementation. Aliquots at selected time points were serially diluted and plated on BHI + Mn plates for CFU enumeration. Survival of strains was recorded over 48 hours. The graphs show the average log10-transformed CFU mean and standard deviations of at least three independent experiments Differences were assessed via two-way ANOVA with Tukey’s post-test (** p ≤ 0.01, ***p ≤ 0.001, **** p ≤ 0.0001). (TIF) [file ppat.1007102.s009.tif]
