# Supplementary material for: Manganese acquisition is essential for virulence of Enterococcus faecalis
Source: PLoS Pathog. 2018 Sep 20;14(9):e1007102. doi: 10.1371/journal.ppat.1007102 (PMC6147510; doi:10.1371/journal.ppat.1007102)
Supplement: S1 Table — (DOCX) [file ppat.1007102.s010.docx]

**Table S1.** Primers used in this study.

| **Primers** | **Sequence*** | **Application** |
| --- | --- | --- |
| 5’ efa Arm 1 | 5’ – CAAGCATACACTTCTAGAGCACCA – 3’ | *efaCBA* deletion |
| 3’ efa Arm 1 | 5’ - GAACGGTGAATTCTTGAACAGCTAAGTTAAA – 3’ | *efaCBA* deletion |
| 5’ efa Arm 2 | 5’ – GAACTGGAATTCAACAAAAATCCA – 3’ | *efaCBA* deletion |
| 3’ efa Arm 2 | 5’ – TTCTATTAGTCTCCCGGGATTATCA – 3' | *efaCBA* deletion |
| 5’ mntH1 Arm 1 | 5’ – CAAGACCCCTGCAGCACA – 3’ | *mntH1* deletion |
| 3’ mntH1 Arm 1 | 5’ - CATAATTAAGCATGCTTTCCGTTTTC – 3’ | *mntH1* deletion |
| 5’ mntH1 Arm 2 | 5’ – TTATGGCATGCTAACTGGTTTAACG – 3’ | *mntH1* deletion |
| 3’ mntH1 Arm 2 | 5’ – CATCGTGCGAATTCGTTCATCATAG – 3’ | *mntH1* deletion |
| 5’ mntH2 Arm 1 | 5’ – GTAATCTAGAACATAAATTAAACAAC – 3’ | *mntH2* deletion |
| 3’ mntH2 Arm 1 | 5’ – ATGTTCTTCTGAATTCTGCAAT – 3’ | *mntH2* deletion |
| 5’ mntH2 Arm 2 | 5’ – CGTTAGCCGAGAATTCTGCCTATG – 3’ | *mntH2* deletion |
| 3’ mntH2 Arm 2 | 5’ – GAAAATCCCGGGCAAGAGAAAG – 3’ | *mntH2* deletion |
| efaCBA Fwd | 5’ – CTGATGGATCC*TTAGTTAGTTAG***AGGAGG** AATTTCATGAGAAAAAGCTTTAACTTAGCTG – 3’ | *efaCBA* complement |
| efaCBA Rev | 5’ – GAGGAAATTGTGGCTCGAGTAAT – 3’ | *efaCBA* complement |
| mntH1 Fwd | 5’ – GAACACTGCAG*TTAGTTAGTTAG***AGGAGG**  GATGTTGGATGAAAGAAAAGA – 3’ | *mntH1* complement |
| mntH1 Rev | 5’ - GTATTTCATCTTTCCTATTCTAGAATTTCTTACG – 3’ | *mntH1* complement |
| mntH2 Fwd | 5’ – TTTTACTGCAG*TTAGTTAGTTAG***AGGAGG**  AATTGAATTGCAGAATTCAGAAC – 3’ | *mntH2* complement |
| mntH2 Rev | 5’ - GAAATGCTTTTAACGCATGCGGCG – 3’ | *mntH2* complement |
| efaA L | 5’ – TGCCGCTTATATTTGGGAAA – 3’ | qRT-PCR |
| efaA R | 5’ – CGCCTTCTGTTCCTTCTTTG – 3’ | qRT-PCR |
| mntH1 L | 5’ – GAGAAAGCCAAAGCAATTCG – 3’ | qRT-PCR |
| mntH1 R | 5’ – TTGACCCGAAGCCAGTAAAG – 3’ | qRT-PCR |
| mntH2 L | 5’ – CCGTGTTGAAATGGGTGAAC – 3’ | qRT-PCR |
| mntH2 R | 5’ – AATTCCACAACCGTCCAAAC – 3’ | qRT-PCR |
| sodA L | 5’ – CAGCGATTGAAAAACATCCA – 3’ | qRT-PCR |
| sodA R | 5’ – TTCATCAAAGCTGCCAAATG – 3’ | qRT-PCR |
| Efa 500 F | 5’ – CATTTACAGGAGCATTCGTTG – 3’ | IE strain screening |
| Efa 1300 R | 5’ – TAAGTGGTGGTGAGCAAAC – 3’ | IE strain screening |
| MntH1 Conf F | 5’ – GAAATGTGTGAACAAGATAGATTG – 3’ | IE strain screening |
| MntH1 Conf R | 5’ – CAACTTTTCCAGTCAGCC – 3’ | IE strain screening |
| MntH2 Conf F | 5’ – GCAAAACGAAAGAAGGAATTG – 3’ | IE strain screening |
| MntH2 Conf R | 5’ – CGACTCTTCAACACCAACC – 3’ | IE strain screening |

*****Underlined bases correspond to restriction sites included to aid in the cloning of PCR products.

*Italicized bases correspond to the termination site (in 3 frames) included for cloning into the pTG001 vector.
*Bold bases correspond to the perfect ribosomal binding site included to ensure gene expression from pTG001 constructs.
